# Supplementary material for: Flexible and robust cell type annotation for highly multiplexed tissue images
Source: bioRxiv. 2024 Dec 17:2024.09.12.612510. Originally published 2024 Sep 16. Preprint. [Version 2] doi: 10.1101/2024.09.12.612510 (PMC11429614; doi:10.1101/2024.09.12.612510)
Supplement: Supplement 1 [file media-1.pdf]

Flexible and robust cell type annotation for highly multiplexed tissue images: Supplementary Information

Huangqingbo Sun, Shiqiu Yu, Anna Martinez Casals, Anna Bäckström, Yuxin Lu, Cecilia Lindskog, Matthew Ruffalo, Emma Lundberg, and Robert F. Murphy

December 17, 2024

1 Supplementary tables

Table S1: Sources of datasets used to curate our integrated dataset for training the models.

Deposited data	Source Article	
Human colorectal cancer CODEX image	[10]	Link
Tonsil CODEX image I		Link
Multi-tumor CODEX image		Link
Lymphoma CODEX image	[9]	Link
Human intestine CODEX image	[5]	Link<br>Link
Bone marrow and acute myeloid leukemia CODEX image	[1]	Link
Tonsil CODEX image II	[2]	Link
Classic Hodgkin Lymphoma MIBI<br>Classic Hodgkin Lymphoma CODEX image	[11]	Link
Triple negative breast cancer MIBI-TOF	[6]	Link
Breast cancer progression MIBI-TOF	[8]	Link
Human tuberculosis MIBI-TOF	[7]	Link
Fetally derived extravillous trophoblasts MIBI	[3]	Link
HuBMAP 29-marker CODEX image (spleen, thymus, and lymph node)		Link
Human kidney CODEX image	[4]	Link

Table S2: Hyper-parameter setting of our customized ViT model.

	ViT encoder				Decoder			
Panel	Layer	Hidden size	MLP size	Heads	Layer	Hidden size	MLP size	Heads
Immune Base	12	288	1152	12	6	192	768	8
Immune Extended	12	384	1536	12	6	192	768	8
Immune Full	12	576	2304	12	6	192	768	8
Tissue Structure	12	288	1152	12	6	192	768	8
Nerve Cell	12	144	576	12	4	96	384	4

Table S3: Antibody list multiplexed in each TMA section: all the antibodies were diluted at 1:200 dilution, except CD163 (1:50) and VIM (1:400). The exposure times were set up at 150ms for all the antibodies, except VIM (100ms). Akoya Biosciences is our antibody vendor.

Target	Barcode	Fluorophore	Cat#
b-Actin	BX117	AF750	4450092
ATM	BX083	Atto550	4250095
Bcl2	BX085	AF647	4550089
b-Catenin	BX096	Atto550	4250091
Caveolin	BX086	AF647	4550084
CD3e	BX045	AF647	4550119
CD4	BX003	AF647	4550112
CD8	BX026	Atto550	4250012
CD11c	BX024	AF647	4550114
CD14	BX037	Atto550	4450047
CD20	BX007	AF750	4450018
CD31	BX001	AF750	4450017
CD34	BX025	Atto550	4250057
CD39	BX099	Atto550	4250076
CD40	BX010	AF647	4550064
CD45	BX021	AF647	4550121
CD44	BX005	Atto550	4450041
CD56	BX028	AF647	4550126
CD68	BX015	AF647	4550113
CD107a	BX006	AF647	4550098
CD163	BX069	Atto550	4250079
COL4A1	BX042	AF647	4550122
E-cadherin	BX014	Atto550	4250021
EpCAM	BX091	AF647	4550088
ER (Estrogen receptor)	BX084	Atto550	4250074
GP100 (PMEL)	BX094	AF647	4550091
HIF1a	BX062	AF647	4550069
HistoneH3Phospho	BX030	AF647	4550115
HLA-A	BX029	Atto550	4250100
HLADR	BX033	AF647	4550118
ICOS	BX054	AF647	4550117
IDO1	BX027	AF647	4550123
iNOS	BX023	Atto550	4250073
Keratin5	BX101	Atto550	4250090
Keratin8/18	BX081	AF750	4450082
Ki67	BX047	AF750	4450096
Pancytokeratin	BX019	AF750	4450020
PCNA	BX036	AF647	4550124
PD1	BX046	AF647	4550038
Podoplanin	BX121	Atto550	4250094
SMA	BX013	AF750	4450049
SOX2	BX102	Atto550	4250075
TOX	BX060	Atto550	4250067
Vimentin	BX022	AF750	4450050
VISTA	BX040	Atto550	4250063

Table S4: Tissue included in the TMA blocks to perform the highly multiplexed staining.

Adrenal gland	Cortex	Hippocampus	Parathyroid	Soft tissue
Appendix	Duodenum	Kidney	Placenta	Skin
Bladder	Endometrium, pre-menopausal	Liver	Prostate	Spleen
Bone marrow	Endometrium, post-menopausal	Lung	Rectum	Stomach
Breast	Epididymis	Lymph node	Seminal vesicle	Testis
Bronchus	Esophagus	Nasopharynx	Salivary gland	Thyroid gland
Caudate& cerebellum	Fallopian tube	Oral tissue	Skeletal muscle	Tonsil
Cervix	Gall bladder	Ovary	Small intestine	Vagina
Colon	Heart muscle	Pancreas	Smooth muscle	Vulva/anal

Table S5: Basic Panel: Mapping of Original Labels to Standardized Labels across Datasets

**Dataset Name**	**Original Label**	**Standardized Label**
Breast_cancer_i	OTHER	others/undefined
Breast_cancer_i	CD8T	CD8+ T cells
Breast_cancer_i	CD4T	CD4+ T cells
Breast_cancer_i	BCELL	B cells
Breast_cancer_i	TCELL	Other T cells
Breast_cancer_i	DC	Dendritic Cell
Breast_cancer_i	all others	all other cells
CRC	B cells	B cells
CRC	CD4+ T cells	CD4+ T cells
CRC	CD4+ T cells CD45RO+	CD4+ T cells
CRC	CD4+ T cells GATA3+	CD4+ T cells
CRC	CD8+ T cells	CD8+ T cells
CRC	CD3+ T cells	Other T cells
CRC	Tregs	Other T cells
CRC	CD11c+ DCs	Dendritic Cell
CRC	undefined	others/undefined
CRC	all others	all other cells
EVT	B cells	B cells
EVT	CD4T	CD4+ T cells
EVT	CD8T	CD8+ T cells
EVT	Treg	Other T cells
EVT	DC	Dendritic Cell
EVT	other	others/undefined
EVT	all others	all other cells
Intestine	B	B cells
Intestine	Plasma	B cells
Intestine	CD4+ T cell	CD4+ T cells
Intestine	CD8+ T	CD8+ T cells
Intestine	Treg	Other T cells
Intestine	DC	Dendritic Cell
Intestine	other	others/undefined
Intestine	all others	all other cells
Lymphoma	B	B cells
Lymphoma	PC	B cells
Lymphoma	TFH	CD4+ T cells
Lymphoma	CD4T	CD4+ T cells
Lymphoma	CD4+ T cell	CD4+ T cells
Lymphoma	CD4TNaive	CD4+ T cells
Lymphoma	TTOX	CD8+ T cells
Lymphoma	TTOX_exh	CD8+ T cells

Dataset Name	Original Label	Standardized Label
Lymphoma	TTOXNaive	CD8+ T cells
Lymphoma	Treg	Other T cells
Lymphoma	FDC	Dendritic Cell
Lymphoma	DC	Dendritic Cell
Lymphoma	na	others/undefined
Lymphoma	all others	all other cells
TB	B_cell	B cells
TB	CD4_T	CD4+ T cells
TB	CD8_T	CD8+ T cells
TB	gdT_cell	Other T cells
TB	Treg	Other T cells
TB	CD11c_DC/Mono	Dendritic Cell
TB	CD209_DC	Dendritic Cell
TB	all others	all other cells
cHL_MIBI	B	B cells
cHL_MIBI	CD4 T	CD4+ T cells
cHL_MIBI	CD4 Treg	CD4+ T cells
cHL_MIBI	CD8 T	CD8+ T cells
cHL_MIBI	Treg	Other T cells
cHL_MIBI	DC	Dendritic Cell
cHL_MIBI	Other	others/undefined
cHL_MIBI	all others	all other cells
cHL_CODEX	B	B cells
cHL_CODEX	CD4	CD4+ T cells
cHL_CODEX	CD8	CD8+ T cells
cHL_CODEX	Cytotoxic CD8	CD8+ T cells
cHL_CODEX	TReg	Other T cells
cHL_CODEX	DC	Dendritic Cell
cHL_CODEX	Seg Artifact	others/undefined
cHL_CODEX	Other	others/undefined
cHL_CODEX	all others	all other cells
Bone_Marrow	B-Cells	B cells
Bone_Marrow	Plasma Cells	B cells
Bone_Marrow	Immature_B_Cell	B cells
Bone_Marrow	CD4+ T-Cell	CD4+ T cells
Bone_Marrow	CD4 Treg	CD4+ T cells
Bone_Marrow	CD8+ T-Cell	CD8+ T cells
Bone_Marrow	pDC	Dendritic Cell
Bone_Marrow	Artifact	others/undefined
Bone_Marrow	Undetermined	others/undefined
Bone_Marrow	Autofluorescent	others/undefined
Bone_Marrow	all others	all other cells
AML	B-Cells	B cells
AML	Plasma Cells	B cells
AML	Immature_B_Cell	B cells
AML	CD4+ T-Cell	CD4+ T cells
AML	CD4 Treg	CD4+ T cells
AML	CD8+ T-Cell	CD8+ T cells
AML	pDC	Dendritic Cell
AML	Artifact	others/undefined
AML	Undetermined	others/undefined
AML	Autofluorescent	others/undefined
AML	all others	all other cells

Table S6: Full Panel: Mapping of Original Labels to Standardized Labels across Datasets

Dataset Name	Original Label	Standardized Label
Breast_cancer_i	TUMOR_LUMINAL	others (defined but not included)
Breast_cancer_i	TUMOR_EMT	others (defined but not included)
Breast_cancer_i	TUMOR_ECADCK	others (defined but not included)
Breast_cancer_i	TUMOR_CK5	others (defined but not included)
Breast_cancer_i	ENDO	others (defined but not included)
Breast_cancer_i	OTHER	artifact/not defined
Breast_cancer_i	NORMFIBRO	others (defined but not included)
Breast_cancer_i	NEUT	Granulocyte (neutrophil)
Breast_cancer_i	MYOFIBRO	others (defined but not included)
Breast_cancer_i	MYOEP	others (defined but not included)
Breast_cancer_i	MONODC	Dendritic cell
Breast_cancer_i	MONO	Macrophage
Breast_cancer_i	MAST	Mast cell
Breast_cancer_i	MACS	others (defined but not included)
Breast_cancer_i	FIBRO_VIMonly	others (defined but not included)
Breast_cancer_i	CD8T	CD8 T cell
Breast_cancer_i	CD4T	CD4 T cell
Breast_cancer_i	CAF	others (defined but not included)
Breast_cancer_i	BCELL	B cell
Breast_cancer_i	APC	others (defined but not included)
Breast_cancer_i	TCELL	others (defined but not included)
Breast_cancer_i	IMMUNEOTHER	others (defined but not included)
Breast_cancer_i	DC	Dendritic cell
Lymph_node	B	B cell
Lymph_node	FDC	Dendritic cell
Lymph_node	TTOX	CD8 T cell
Lymph_node	Macro	Macrophage
Lymph_node	na	artifact/not defined
Lymph_node	CD4T	CD4 T cell
Lymph_node	Stromal cells	others (defined but not included)
Lymph_node	TFH	CD4 T cell
Lymph_node	DC	Dendritic cell
Lymph_node	Granulo	Granulocyte (neutrophil)
Lymph_node	Treg	Treg cell
Lymph_node	TPR	others (defined but not included)
Lymph_node	NK	NK cell
Lymph_node	CD4TNaive	CD4 T cell
Lymph_node	TTOX_exh	CD8 T cell
Lymph_node	TTOXNaive	CD8 T cell
Lymph_node	MC	Macrophage
Lymph_node	PC	Plasma cell
Lymph_node	NKT	NK cell
cHL_MIBI	Other	artifact/not defined
cHL_MIBI	CD4 T	CD4 T cell
cHL_MIBI	Endothelial	others (defined but not included)
cHL_MIBI	M1	Macrophage
cHL_MIBI	B	B cell
cHL_MIBI	M2	M2 Macrophage
cHL_MIBI	NK	NK cell
cHL_MIBI	DC	Dendritic cell
cHL_MIBI	CD4 Treg	Treg cell
cHL_MIBI	Neutrophil	Granulocyte (neutrophil)

Dataset Name	Original Label	Standardized Label
cHL_MIBI	CD8 T	CD8 T cell
cHL_MIBI	Tumor	others (defined but not included)
cHL_CODEX	B	B cell
cHL_CODEX	DC	Dendritic cell
cHL_CODEX	NK	NK cell
cHL_CODEX	Monocyte	Macrophage
cHL_CODEX	CD4	CD4 T cell
cHL_CODEX	Lymphatic	others (defined but not included)
cHL_CODEX	CD8	CD8 T cell
cHL_CODEX	M1	Macrophage
cHL_CODEX	Seg Artifact	artifact/not defined
cHL_CODEX	Endothelial	others (defined but not included)
cHL_CODEX	Mast	Mast cell
cHL_CODEX	Tumor	others (defined but not included)
cHL_CODEX	Neutrophil	Granulocyte (neutrophil)
cHL_CODEX	M2	M2 Macrophage
cHL_CODEX	TReg	Treg cell
cHL_CODEX	Cytotoxic CD8	CD8 T cell
cHL_CODEX	Epithelial	others (defined but not included)
cHL_CODEX	Other	artifact/not defined
CRC	granulocytes	Granulocyte (neutrophil)
CRC	vasculature	others (defined but not included)
CRC	CD4+ T cells CD45RO+	CD4 T cell
CRC	tumor cells	others (defined but not included)
CRC	stroma	others (defined but not included)
CRC	CD68+CD163+ macrophages	M2 Macrophage
CRC	adipocytes	others (defined but not included)
CRC	Plasma cells	Plasma cell
CRC	CD8+ T cells	CD8 T cell
CRC	dirt	artifact/not defined
CRC	Tregs	Treg cell
CRC	CD4+ T cells	CD4 T cell
CRC	CD11c+ DCs	Dendritic cell
CRC	B cells	B cell
CRC	CD11b+CD68+ macrophages	Macrophage
CRC	smooth muscle	others (defined but not included)
CRC	undefined	artifact/not defined
CRC	tumor cells / immune cells	others (defined but not included)
CRC	immune cells / vasculature	others (defined but not included)
CRC	immune cells	others (defined but not included)
CRC	NK cells	NK cell
CRC	nerves	others (defined but not included)
CRC	CD68+ macrophages GzmB+	Macrophage
CRC	CD68+ macrophages	Macrophage
CRC	lymphatics	others (defined but not included)
CRC	CD11b+ monocytes	Macrophage
CRC	CD4+ T cells GATA3+	CD4 T cell
CRC	CD163+ macrophages	M2 Macrophage
CRC	CD3+ T cells	others (defined but not included)
EVT	Mac2a	M2 Macrophage
EVT	other	artifact/not defined
EVT	NK1	NK cell
EVT	Fibroblasts	others (defined but not included)
EVT	NKT	NK cell
EVT	Endothelial	others (defined but not included)

Dataset Name	Original Label	Standardized Label
EVT	Myofibroblasts	others (defined but not included)
EVT	Mac1a	Macrophage
EVT	EVT1a	others (defined but not included)
EVT	Mac1b	Macrophage
EVT	CD8T	CD8 T cell
EVT	EVT1b	others (defined but not included)
EVT	Mac2c	M2 Macrophage
EVT	NK2	NK cell
EVT	muscle	others (defined but not included)
EVT	NK3	NK cell
EVT	EVT2	others (defined but not included)
EVT	Mac2b	M2 Macrophage
EVT	DC	Dendritic cell
EVT	Glandular	others (defined but not included)
EVT	CD4T	CD4 T cell
EVT	EVT1c	others (defined but not included)
EVT	Placental_Mac	M2 Macrophage
EVT	NK4	NK cell
EVT	Mast	Mast cell
EVT	Treg	Treg cell

Table S7: Extended Panel: Mapping of Original Labels to Standardized Labels across Datasets

Dataset Name	Original Label	Standardized Label
TB	CD163_Mac	M2 Macrophage
TB	CD68_Mac	Macrophage
TB	CD16_CD14_Mono	Macrophage
TB	CD4_T	CD4+ T cell
TB	CD14_Mono	Macrophage
TB	fibroblast	other (defined but not included)
TB	endothelial	other (defined but not included)
TB	Treg	Treg cell
TB	CD11c_DC/Mono	Dendritic cell
TB	imm_other	other (defined but not included)
TB	CD11b/c_CD206_Mac/Mono	Macrophage
TB	CD8_T	CD8+ T cell
TB	B_cell	B cell
TB	neutrophil	Granulocyte
TB	CD206_Mac	M2 Macrophage
TB	mast	Mast cell
TB	epithelial	other (defined but not included)
TB	giant_cell	other (defined but not included)
TB	gdT_cell	other (defined but not included)
TB	CD209_DC	Dendritic cell
Intestine	NK	NK cell
Intestine	Enterocyte	other (defined but not included)
Intestine	MUC1+ Enterocyte	other (defined but not included)
Intestine	TA	other (defined but not included)
Intestine	CD66+ Enterocyte	other (defined but not included)
Intestine	Paneth	other (defined but not included)
Intestine	Cycling TA	other (defined but not included)
Intestine	Goblet	other (defined but not included)
Intestine	Smooth muscle	other (defined but not included)
Intestine	M1 Macrophage	Macrophage
Intestine	Neuroendocrine	other (defined but not included)
Intestine	Stroma	other (defined but not included)
Intestine	Nerve	other (defined but not included)
Intestine	ICC	other (defined but not included)
Intestine	Lymphatic	other (defined but not included)
Intestine	Plasma	Plasma cell
Intestine	CD4+ T cell	CD4+ T cell
Intestine	M2 Macrophage	M2 Macrophage
Intestine	DC	Dendritic cell
Intestine	B	B cell
Intestine	CD57+ Enterocyte	other (defined but not included)
Intestine	CD8+ T	CD8+ T cell
Intestine	Neutrophil	Granulocyte
Intestine	Endothelial	other (defined but not included)
Intestine	Cycling TA	other (defined but not included)
Intestine	CD7+ Immune	other (defined but not included)
Bone Marrow	SEC	other (defined but not included)
Bone Marrow	Intermediate Myeloid	other (defined but not included)
Bone Marrow	Erythroblast	other (defined but not included)
Bone Marrow	Mature Myeloid	other (defined but not included)
Bone Marrow	Erythroid	other (defined but not included)
Bone Marrow	Plasma Cells	Plasma cell

Dataset Name	Original Label	Standardized Label
Bone Marrow	NPM1 Mutant Blast	other (defined but not included)
Bone Marrow	CD8+ T-Cell	CD8+ T cell
Bone Marrow	B-Cells	B cell
Bone Marrow	Early Myeloid Progenitor	other (defined but not included)
Bone Marrow	Adipocyte	other (defined but not included)
Bone Marrow	CD4+ T-Cell	CD4+ T cell
Bone Marrow	Macrophages	Macrophage
Bone Marrow	AEC	other (defined but not included)
Bone Marrow	Immature_B_Cell	B cell
Bone Marrow	GATA1pos_Mks	other (defined but not included)
Bone Marrow	Monocytes	Macrophage
Bone Marrow	pDC	Dendritic cell
Bone Marrow	Endosteal	other (defined but not included)
Bone Marrow	Adipo-MSC	other (defined but not included)
Bone Marrow	Non-Classical Monocyte	Macrophage
Bone Marrow	GMP	other (defined but not included)
Bone Marrow	HSPC	other (defined but not included)
Bone Marrow	THY1+ MSC	other (defined but not included)
Bone Marrow	VSMC	other (defined but not included)
Bone Marrow	CD34+ CD61+	other (defined but not included)
Bone Marrow	GATA1neg_Mks	other (defined but not included)
Bone Marrow	SPINK2+ HSPC	other (defined but not included)
Bone Marrow	Artifact	artifact/not defined
Bone Marrow	Undetermined	artifact/not defined
Bone Marrow	Autofluorescent	artifact/not defined
Bone Marrow	CD44+ Undetermined	other (defined but not included)
Bone Marrow	MEP/Early Erythroblast	other (defined but not included)
Bone Marrow	GMP/Myeloblast	other (defined but not included)
Bone Marrow	HSC	other (defined but not included)
Bone Marrow	CLP	other (defined but not included)
Bone Marrow	Schwann Cells	other (defined but not included)

Table S8: Structure Panel: Mapping of Original Labels to Standardized Labels across Datasets

**Dataset Name**	**Original Label**	**Standardized Label**
CRC	granulocytes	all the other cell types
CRC	vasculature	endothelial
CRC	CD4+ T cells CD45RO+	all the other cell types
CRC	tumor cells	tumor
CRC	stroma	stroma
CRC	CD68+CD163+ macrophages	all the other cell types
CRC	adipocytes	all the other cell types
CRC	plasma cells	all the other cell types
CRC	CD8+ T cells	all the other cell types
CRC	dirt	undefined
CRC	Tregs	all the other cell types
CRC	CD4+ T cells	all the other cell types
CRC	CD11c+ DCs	all the other cell types
CRC	B cells	all the other cell types
CRC	CD11b+CD68+ macrophages	all the other cell types
CRC	smooth muscle	smooth muscle
CRC	undefined	undefined
CRC	tumor cells / immune cells	tumor
CRC	immune cells / vasculature	endothelial
CRC	immune cells	all the other cell types
CRC	NK cells	all the other cell types
CRC	nerves	all the other cell types
CRC	CD68+ macrophages GzmB+	all the other cell types
CRC	CD68+ macrophages	all the other cell types
CRC	lymphatics	smooth muscle
CRC	CD11b+ monocytes	all the other cell types
CRC	CD4+ T cells GATA3+	all the other cell types
CRC	CD163+ macrophages	all the other cell types
CRC	CD3+ T cells	all the other cell types
cHL_CODEX	B	all the other cell types
cHL_CODEX	DC	all the other cell types
cHL_CODEX	NK	all the other cell types
cHL_CODEX	Monocyte	all the other cell types
cHL_CODEX	CD4	all the other cell types
cHL_CODEX	Lymphatic	smooth muscle
cHL_CODEX	CD8	all the other cell types
cHL_CODEX	M1	all the other cell types
cHL_CODEX	Seg Artifact	undefined
cHL_CODEX	Endothelial	endothelial
cHL_CODEX	Mast	all the other cell types
cHL_CODEX	Tumor	tumor
cHL_CODEX	Neutrophil	all the other cell types
cHL_CODEX	M2	all the other cell types
cHL_CODEX	TReg	all the other cell types
cHL_CODEX	Cytotoxic CD8	all the other cell types
cHL_CODEX	Epithelial	epithelial
cHL_CODEX	Other	undefined
Breast Cancer	TUMOR_LUMINAL	tumor
Breast Cancer	TUMOR_EMT	tumor
Breast Cancer	TUMOR_ECADCK	tumor
Breast Cancer	TUMOR_CK5	tumor
Breast Cancer	ENDO	endothelial

**Dataset Name**	**Original Label**	**Standardized Label**
Breast Cancer	OTHER	undefined
Breast Cancer	NORMFIBRO	stroma
Breast Cancer	NEUT	all the other cell types
Breast Cancer	MYOFIBRO	smooth muscle
Breast Cancer	MYOEP	epithelial
Breast Cancer	MONODC	all the other cell types
Breast Cancer	MONO	all the other cell types
Breast Cancer	MAST	all the other cell types
Breast Cancer	MACS	undefined
Breast Cancer	FIBRO_VIMonly	stroma
Breast Cancer	CD8T	all the other cell types
Breast Cancer	CD4T	all the other cell types
Breast Cancer	CAF	stroma
Breast Cancer	BCELL	all the other cell types
Breast Cancer	APC	all the other cell types
Breast Cancer	TCELL	all the other cell types
Breast Cancer	IMMUNEOTHER	all the other cell types
Breast Cancer	DC	all the other cell types
EVT	Mac2a	all the other cell types
EVT	other	undefined
EVT	NK1	all the other cell types
EVT	Fibroblasts	stroma
EVT	NKT	all the other cell types
EVT	Endothelial	endothelial
EVT	Myofibroblasts	smooth muscle
EVT	Mac1a	all the other cell types
EVT	EVT1a	all the other cell types
EVT	Mac1b	all the other cell types
EVT	CD8T	all the other cell types
EVT	EVT1b	all the other cell types
EVT	Mac2c	all the other cell types
EVT	NK2	all the other cell types
EVT	muscle	smooth muscle
EVT	NK3	all the other cell types
EVT	EVT2	all the other cell types
EVT	Mac2b	all the other cell types
EVT	DC	all the other cell types
EVT	Glandular	epithelial
EVT	CD4T	all the other cell types
EVT	EVT1c	all the other cell types
EVT	Placental_Mac	all the other cell types
EVT	NK4	all the other cell types
EVT	Mast	all the other cell types
EVT	Treg	all the other cell types

Table S9: Nerve Panel: Mapping of Original Labels to Standardized Labels across Datasets

**Dataset Name**	**Original Label**	**Standardized Label**
Tonsil_Unlabeled	Innate	all the other cell types
Tonsil_Unlabeled	PDPN	all the other cell types
Tonsil_Unlabeled	Endothelial	all the other cell types
Tonsil_Unlabeled	B cell	all the other cell types
Tonsil_Unlabeled	T cell	all the other cell types
Tonsil_Unlabeled	Squamous epithelial	all the other cell types
Tonsil_Unlabeled	Stromal	all the other cell types
Tonsil_Unlabeled	smooth muscle	all the other cell types
Tonsil_Unlabeled	Plasma Cell	all the other cell types
Tonsil_Unlabeled	Nerve	nerve
CRC	granulocytes	all the other cell types
CRC	vasculature	all the other cell types
CRC	CD4+ T cells CD45RO+	all the other cell types
CRC	tumor cells	all the other cell types
CRC	stroma	all the other cell types
CRC	CD68+CD163+ macrophages	all the other cell types
CRC	adipocytes	all the other cell types
CRC	plasma cells	all the other cell types
CRC	CD8+ T cells	all the other cell types
CRC	dirt	undefined
CRC	Tregs	all the other cell types
CRC	CD4+ T cells	all the other cell types
CRC	CD11c+ DCs	all the other cell types
CRC	B cells	all the other cell types
CRC	CD11b+CD68+ macrophages	all the other cell types
CRC	smooth muscle	all the other cell types
CRC	undefined	undefined
CRC	tumor cells / immune cells	all the other cell types
CRC	immune cells / vasculature	all the other cell types
CRC	immune cells	all the other cell types
CRC	NK cells	all the other cell types
CRC	nerves	nerve
CRC	CD68+ macrophages GzmB+	all the other cell types
CRC	CD68+ macrophages	all the other cell types
CRC	lymphatics	all the other cell types
CRC	CD11b+ monocytes	all the other cell types
CRC	CD4+ T cells GATA3+	all the other cell types
CRC	CD163+ macrophages	all the other cell types
CRC	CD3+ T cells	all the other cell types
Intestine	NK	all the other cell types
Intestine	Enterocyte	all the other cell types
Intestine	MUC1+ Enterocyte	all the other cell types
Intestine	TA	all the other cell types
Intestine	CD66+ Enterocyte	all the other cell types
Intestine	Paneth	all the other cell types
Intestine	Cycling TA	all the other cell types
Intestine	Goblet	all the other cell types
Intestine	Smooth muscle	all the other cell types
Intestine	M1 Macrophage	all the other cell types
Intestine	Neuroendocrine	nerve
Intestine	Stroma	all the other cell types
Intestine	Nerve	nerve

**Dataset Name**	**Original Label**	**Standardized Label**
Intestine	ICC	all the other cell types
Intestine	Lymphatic	all the other cell types
Intestine	Plasma	all the other cell types
Intestine	CD4+ T cell	all the other cell types
Intestine	M2 Macrophage	all the other cell types
Intestine	DC	all the other cell types
Intestine	B	all the other cell types
Intestine	CD57+ Enterocyte	all the other cell types
Intestine	CD8+ T	all the other cell types
Intestine	M2 Macrophage	all the other cell types
Intestine	Neutrophil	all the other cell types
Intestine	Endothelial	all the other cell types
Intestine	Cycling TA	all the other cell types
Intestine	CD7+ Immune	all the other cell types
Intestine	Undefined (NaN)	undefined

2 Supplementary figures

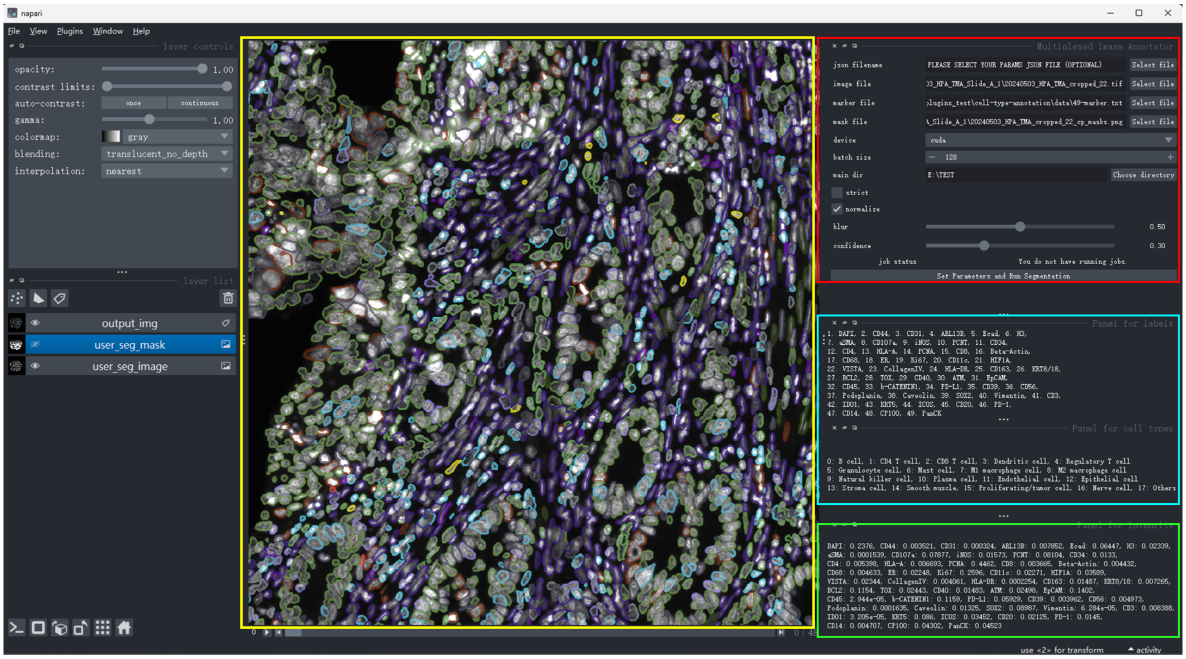

Figure S1: Napari-based visualization of our cell type annotator. The main Napari build-in viewer (yellow box) shows the original tissue image, cell segmentation, and our annotation results in three layers; the panel in red box is for user to required file paths and hyperparameters; below, the panel in cyan lists marker and cell type names; when the user clicks any cells on the cell segmentation layer in the main viewer, the green panel will show the cell-level marker expression intensity of that cell.

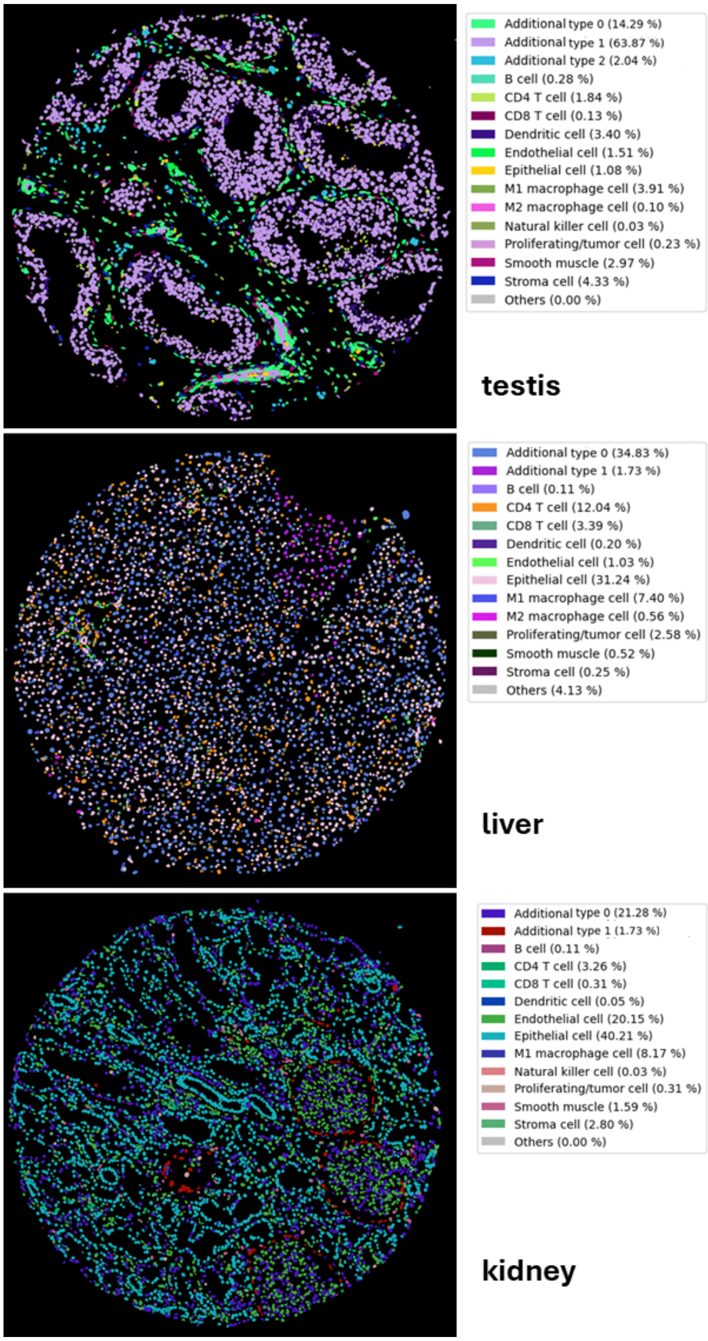

Figure S2: RIBCA enables the identification of tissue-specific cell types through unsupervised clustering of cells that were not previously annotated to 18 known cell types. We identified interstitial cells (additional subtypes 0 and 1) and epididymal duct (additional subtype 2) in testis tissue; kupffer cell (additional subtype 1) in liver tissue.

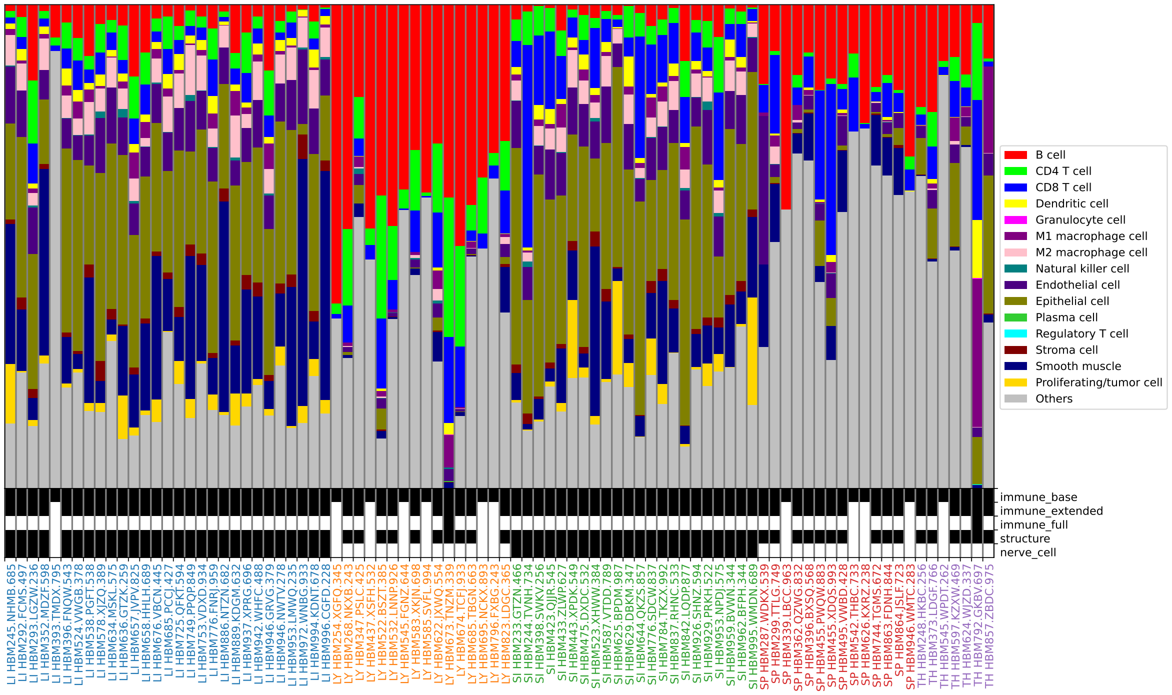

Figure S3: Analysis of individual HuBMAP CODEX datasets. The tissue and the HuBMAP dataset ID are shown along the bottom (the corresponding datasets can be viewed and downloaded from the HuBMAP portal using the IDs). Rows labeled with the names of RIBCA panels show in black those datasets that have sufficient markers to run that panel (note that not all markers are needed since some can be imputed). The fraction of cell types assigned are shown. Note the relative consistency of results within a tissue for those datasets with at least the immune_extended and structure panels (and the fluctuations among the others). The averages for those are shown in Figure 3G.

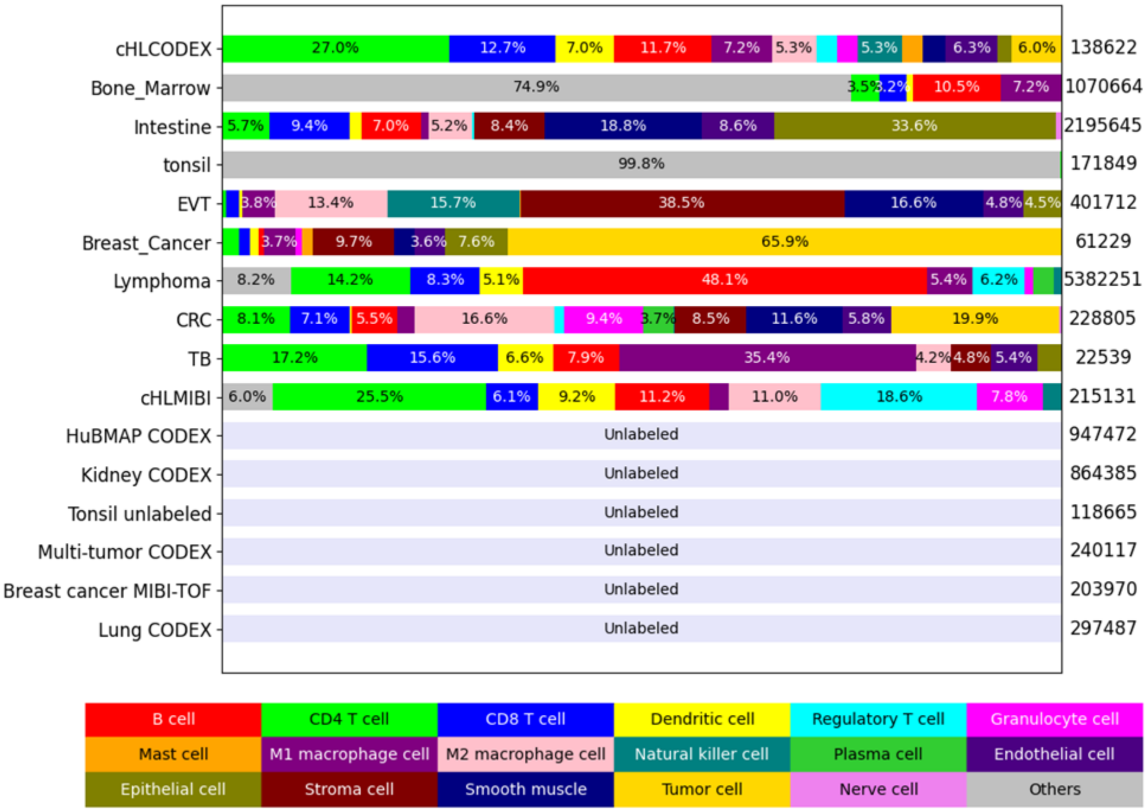

Figure S4: Statistics (cell type distributions and number of cells) of multiple public multiplexed imaging datasets used to curate our dataset to train the models. We reassigned existing annotations to 18 consistent cell types. This set of cell types is at an appropriate granularity and compatible with most existing annotations.

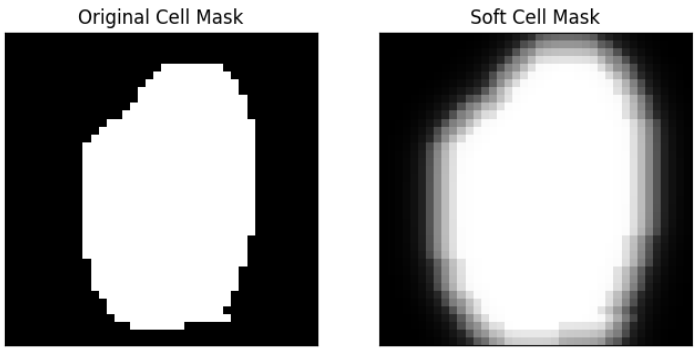

Figure S5: An example of applying soft-masking to a cell segmentation mask.

References

[1] S. Bandyopadhyay, M. P. Duffy, K. J. Ahn, J. H. Sussman, M. Pang, D. Smith, G. Duncan, I. Zhang, J. Huang, Y. Lin, et al. Mapping the cellular biogeography of human bone marrow niches using single-cell transcriptomics and proteomic imaging. *Cell*, 187(12):3120–3140, 2024.

[2] S. Black, D. Phillips, J. W. Hickey, J. Kennedy-Darling, V. G. Venkataraaman, N. Samusik, Y. Goltsev, C. M. Schürch, and G. P. Nolan. Codex multiplexed tissue imaging with dna-conjugated antibodies. *Nature protocols*, 16(8):3802–3835, 2021.

[3] S. Greenbaum, I. Averbukh, E. Soon, G. Rizzuto, A. Baranski, N. F. Greenwald, A. Kagel, M. Bosse, E. G. Jaswa, Z. Khair, et al. A spatially resolved timeline of the human maternal–fetal interface. *Nature*, 619(7970):595–605, 2023.

[4] J. Hansen, R. Sealfon, R. Menon, M. T. Eadon, B. B. Lake, B. Steck, K. Anjani, S. Parikh, T. K. Sigdel, G. Zhang, et al. A reference tissue atlas for the human kidney. *Science advances*, 8(23): eabn4965, 2022.

[5] J. W. Hickey, W. R. Becker, S. A. Nevins, A. Horning, A. E. Perez, C. Zhu, B. Zhu, B. Wei, R. Chiu, D. C. Chen, et al. Organization of the human intestine at single-cell resolution. *Nature*, 619(7970):572–584, 2023.

[6] L. Keren, M. Bosse, D. Marquez, R. Angoshtari, S. Jain, S. Varma, S.-R. Yang, A. Kurian, D. Van Valen, R. West, et al. A structured tumor-immune microenvironment in triple negative breast cancer revealed by multiplexed ion beam imaging. *Cell*, 174(6):1373–1387, 2018.

[7] E. F. McCaffrey, M. Donato, L. Keren, Z. Chen, A. Delmastro, M. B. Fitzpatrick, S. Gupta, N. F. Greenwald, A. Baranski, W. Graf, et al. The immunoregulatory landscape of human tuberculosis granulomas. *Nature immunology*, 23(2):318–329, 2022.

[8] T. Risom, D. R. Glass, I. Averbukh, C. C. Liu, A. Baranski, A. Kagel, E. F. McCaffrey, N. F. Greenwald, B. Rivero-Gutiérrez, S. H. Strand, et al. Transition to invasive breast cancer is associated with progressive changes in the structure and composition of tumor stroma. *Cell*, 185 (2):299–310, 2022.

[9] T. Roider, M. A. Baertsch, D. Fitzgerald, H. Voehringer, B. J. Brinkmann, F. Czernilofsky, M. Knoll, L. Llaó-Cid, A. Mathioudaki, B. Faßbender, et al. Multimodal and spatially resolved profiling identifies distinct patterns of t cell infiltration in nodal b cell lymphoma entities. *Nature Cell Biology*, pages 1–12, 2024.

[10] C. M. Schürch, S. S. Bhate, G. L. Barlow, D. J. Phillips, L. Noti, I. Zlobec, P. Chu, S. Black, J. Demeter, D. R. McIlwain, et al. Coordinated cellular neighborhoods orchestrate antitumoral immunity at the colorectal cancer invasive front. *Cell*, 182(5):1341–1359, 2020.

[11] M. Shaban, Y. Bai, H. Qiu, S. Mao, J. Yeung, Y. Y. Yeo, V. Shanmugam, H. Chen, B. Zhu, J. L. Weirather, et al. Maps: Pathologist-level cell type annotation from tissue images through machine learning. *Nature Communications*, 15(1):28, 2024.
